# Injuries of the posteromedial bundle of the posterior cruciate ligament after knee hyperextension trauma: A new clinical entity based on an original case series

**DOI:** 10.1002/jeo2.12052

**Published:** 2024-07-05

**Authors:** Caroline Mouton, Maximiliano Ibañez, Felix Hoffmann, Joan Carles Monllau, Romain Seil

**Affiliations:** ^1^ Department of Orthopaedic Surgery Centre Hospitalier Luxembourg—Clinique d'Eich Luxembourg Luxembourg; ^2^ Luxembourg Institute of Research in Orthopaedics, Sports Medicine and Science (LIROMS) Luxembourg Luxembourg; ^3^ ICATME, Hospital Universitari Dexeus, UAB Barcelona Spain; ^4^ Department of Orthopaedic Surgery, Parc de Salut Mar, Hospital de la Esperanza Universitat Autònoma de Barcelona Barcelona Spain; ^5^ Human Motion, Orthopaedics, Sports Medicine and Digital Methods (HOSD), Luxembourg Institute of Health (LIH) Luxembourg Luxembourg

**Keywords:** hyperextension, partial tears, persistent knee pain, posteromedial bundle, posterior cruciate ligament

## Abstract

**Purpose:**

This original case series aims to describe an uncommon triad of clinical signs in patients presenting with persistent pain and inability to resume physical activities after knee hyperextension trauma.

**Methods:**

Patient history, clinical examination, arthroscopic findings and investigations of 12 patients who consulted with the senior author are presented.

**Results:**

Twelve patients (seven males/five females) presented with persistent pain after knee hyperextension trauma either in sport or a traffic accident. They had a median age of 18.5 and a median body mass index of 23 kg/m^2^. All had medical visits and at least one magnetic resonance imaging (MRI) before visiting the senior author's institution but the cause of their problems could not be explained. The clinical examination of the injured knee appeared normal except for an uncommon triad of clinical signs with the knee close to extension: (1) a grade 1+ anterior–posterior laxity around 10–20° of knee flexion with a firm end‐point (pseudo‐Lachman sign), (2) a grade 1+ tibiofemoral step‐off sign with a posterior drawer at 10–20° of knee flexion and (3) an increased knee hyperextension compared to the contralateral side. Arthroscopy of eight patients confirmed the pseudo‐Lachman sign with a grade I posterior drawer close to knee extension, normal posterior laxity at 90° of knee flexion and an intact anterior cruciate ligament.

**Conclusion:**

Patients displayed an increased hyperextension and posterior laxity close to knee extension which normalised at 90° of knee flexion. In patients with a history of knee hyperextension trauma associated with persistent pain, inability to resume physical activities, inconclusive MRIs and a standard clinical examination, clinicians should consider extending their investigations with the knee close to extension to identify this clinical triad consistent with a lesion to the posteromedial bundle of the posterior cruciate ligament.

**Level of evidence:**

Level IV.

**Abbreviations**
ACL: anterior cruciate ligament
ALB: anterolateral bundle
BME: bone marrow oedema
BMI: body mass index
CT: computer tomography
MRI: magnetic resonance imaging
PCL: posterior cruciate ligament
PMB: posteromedial bundle
POL: oblique popliteal ligament
PTT: posterior tibial translation
SSD: side‐to‐side difference

## INTRODUCTION

Hyperextension knee injuries are frequent in athletes, most often occurring without contact, on a fully extended leg during landing or a forward fall on a fixed foot [7, 12, 13, 21]. Previous biomechanical studies suggested that the anterior cruciate ligament (ACL), posterior cruciate ligament (PCL) and oblique popliteal ligament (POL) may be injured during such trauma [1, 4, 6, 15]. In a retrospective diagnostic case series of 25 patients with a knee magnetic resonance imaging (MRI) within 1 year after a hyperextension injury, Ali et al. [1] observed a high rate of injuries to the posterior capsule (52%, *n* = 13), the PCL (40%, *n* = 10) and the ACL (40%, *n* = 10). Interestingly, PCL injuries were all partial thickness tears. Unfortunately, the authors did not further investigate whether the anterolateral bundle (ALB) and/or the posteromedial bundle (PMB) were involved, nor did they confirm the extent of structural tissue damage under clinical examination or arthroscopy.

The diagnosis of partial PCL injuries currently remains a challenge. Patients may indeed present with vague, unspecific, subacute or chronic symptoms of minimal pain and swelling [10, 11, 12]. The use of MRI is also limited to accurately determine the presence and extent of a PCL injury [18, 20] as it is common that the PCL regains continuity over time [5]. To date, no clear diagnostic strategy thus exists to efficiently identify partial PCL ruptures.

As the majority of PCL injuries occur in knee flexion and because the ALB is known to be the primary restraint of posterior tibial translation (PTT) from 30° to 120° of knee flexion [2], the ALB is thought to be more commonly injured than the PMB. Less is known about other injury mechanisms occurring at lower angle of knee flexion or in hyperextension injuries. While both bundles are recognised to play a significant role in resisting PTT at all degrees of knee flexion, the PMB is known to have a supplemental role at low flexion angles [11]. As described by Amis et al. [3], the PMB is tight and aligned in a proximal–distal direction in the extended knee. This prevents the PMB to act as a primary restraint of the posterior tibial drawer at low degrees of knee flexion. It can however resist hyperextension so that it may be overstretched or injured during knee hyperextension trauma.

The aim of this original case series is to describe an uncommon triad of clinical signs observed in patients presenting with unresolved persistent pain and inability to resume physical activities after knee hyperextension trauma, which could be consistent with a partial PCL injury of the PMB.

## CASE SERIES

Twelve patients (five females and seven males) that sustained either a sport (*n* = 11) or a traffic (*n* = 1) accident with a hyperextension mechanism, referred for a consultation with the senior author, are presented. As data were anonymously collected from the medical records by the team of clinicians taking care of these patients, data collection was performed in accordance with the ethical standards of the institution and did not require prior approval.

### Patient history

Demographic and injury data as well as all medical information already available at the time of the first medical visit with the senior author are displayed in Table 1 for each patient in the chronological order of presentation. Patients were relatively young with a median age of 18.5 (Quartile 1–3: 16.3–26.0) and displayed a body mass index within the normal range (median: 23 kg/m^2^; Quartile 1–3: 20–24).

**Table 1:** Demographics, injury data and available medical information at first medical visit with the senior author.

Patient	1	2	3	4	5
Sex	Male	Male	Female	Male	Female
Age (years)	17	24	14	17	20
BMI (kg/m^2^)	22.4	24.8	20.3	22.6	23.4
Injured knee	Left	Left	Left	Right	Left
Activity at injury	Basketball	Football	Football	Basketball	Volleyball
Mechanism of injury	Hyperextension*2—contact with another player and while landing 4 months apart	Hyperextension—direct trauma in front of the knee	Noncontact hyperextension	Hyperextension—direct trauma in front of the knee	Hyperextension *×2—5 months apart
Time since injury (months)	9	<1	3	<1	12
Available information	Two MRIs after each trauma; no findings except an anteromedial bone bruise, functional tests show postural instability and hamstrings deficit (15%), posterior tibial slope of 12°	MRI 2 weeks after the injury; referred for a posterolateral corner injury	2 MRIs after the injury and at 3 months; referred for a medial meniscus tear	MRI 1 week after the injury; referred for posterolateral instability and possible partial PCL lesion	Three MRIs performed after the injury, at 6 and 11 months; no findings
Symptoms	Persistent posteromedial pain, unable to play basketball	Diffuse knee pain, discomfort, lameness without feeling of giving way	Persistent knee pain including at rest, unable to play football	Knee pain, effusion requiring aspiration (30 cc of haematic fluid)	Persistent chronic anterolateral pain, unable to play volleyball

Patient	6	7	8	9	10
Sex	Male	Male	Female	Male	Female
Age (years)	17	51	45	13	13
BMI (kg/m^2^)	22.7	25.7	17.6	20.4	19.5
Injured knee	Left	Left	Left	Right	Left
Activity at injury	Gymnastics	Motorbike accident	Football	Handball	Handball
Mechanism of injury	Hyperextension × 2 while landing from a jump repeated 2 weeks apart	Unknown	Unknown	Hyperextension	Hyperextension—contact with another player
Time since injury (months)	2	96	48	10	2
Available information	Two MRIs 1 and 2 weeks after the injury; referred for a partial ACL lesion	Both knees injured with tibial osteosynthesis, two MRIs 4 years after injury; referred for an isolated medial meniscus tear	Two MRIs after the injury and 3 years later; referred for an alteration of ACL and probably partial PCL	Three MRIs after the injury, at 4 and 10 months; no clear findings	MRI 1 month after the injury; referred for a medial meniscus tear
Symptoms	Initially pain but resolved at first consultation	Persistent knee pain with effusion, physical work with 3 sick leaves per year	Persistent knee pain, unable to play football	Persistent knee pain despite several conservative treatment attempts, unable to play handball	Persistent anterior knee pain

Patient		11		12	
Sex		Male		Female	
Age (years)		32		22	
BMI (kg/m^2^)		24.9		Unknown	
Injured knee		Left		Right	
Activity at injury		Basketball		Football	
Mechanism of injury		Hyperextension without contact		Hyperextension with contact	
Time since injury (months)		<1		4	
Available information		MRI: No abnormality observed to cruciate ligaments CT scan: Nondisplaced impaction fracture		MRIs 2 months after the injury; flat PCL, referred due to suspicion of partial PCL tear	
Symptoms		Walk with scrutches. Swollen knee		Persistent knee pain	

*Note*: Patient are presented in the chronological order. They presented with persistent pain after hyperextension trauma. They also already underwent MRIs and medical visits before visiting the authors' institution. In none of them the cause of their problems could be explained.

Abbreviations: ACL, anterior cruciate ligament; CT, computer tomography; MRI, magnetic resonance imaging; PCL, posterior cruciate ligament.

The common factors between these patients are that they consulted for unresolved persistent pain at a median time of 4 months (Quartile 1: 2–Quartile 3: 11 months) after at least one knee hyperextension trauma. All had undergone an MRI (eight patients even two) and medical visits before visiting the authors' institution. In none of them, the cause of their problems could be explained. None of them reported any experience of knee instability.

### Clinical examination

**Figure 1:**
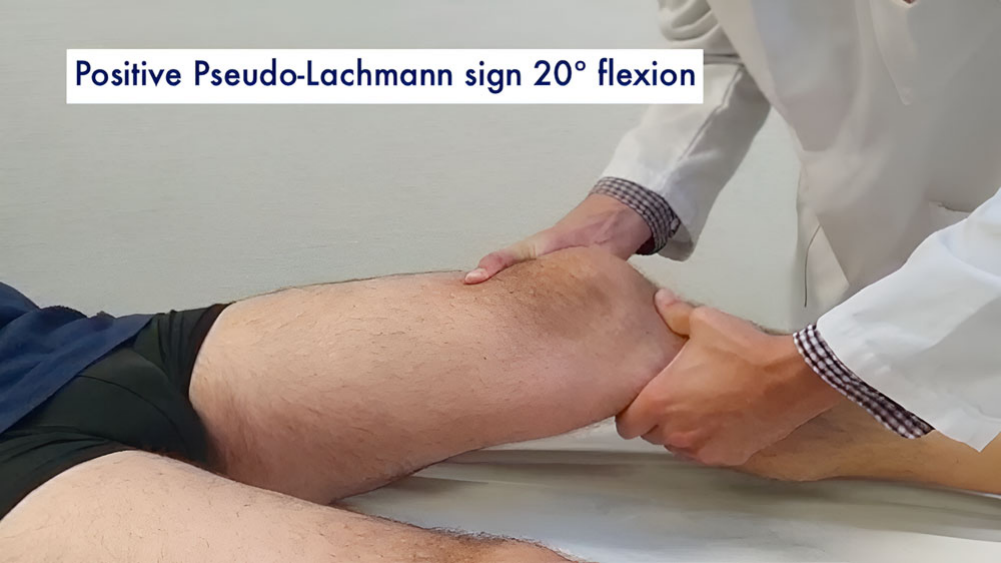
Grade 1+ anterior–posterior laxity around 10–20° of knee flexion with a firm endpoint (pseudo‐Lachman sign).

**Figure 2:**
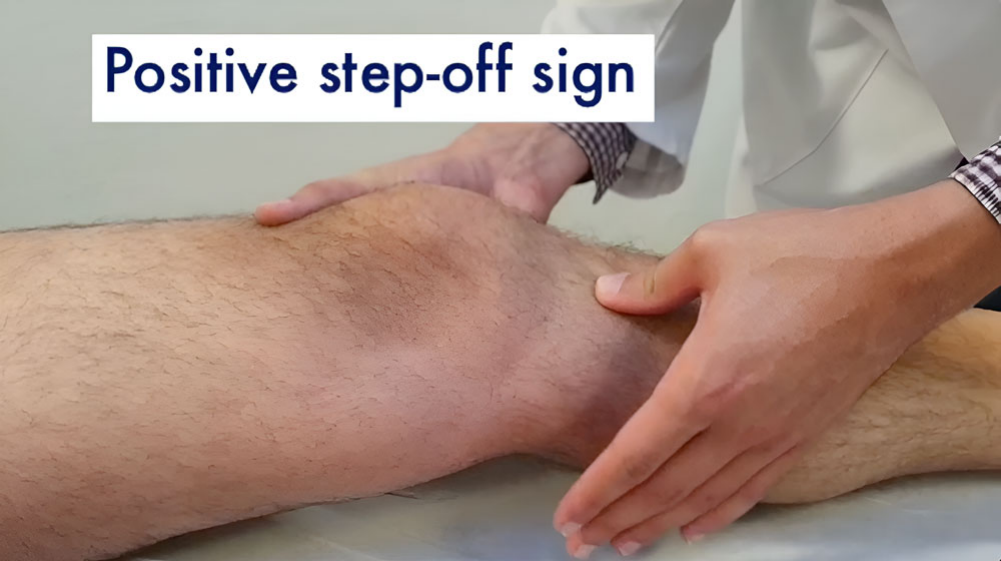
Grade 1+ tibiofemoral step‐off sign with a posterior drawer at 10–20° of knee flexion.

**Figure 3:**
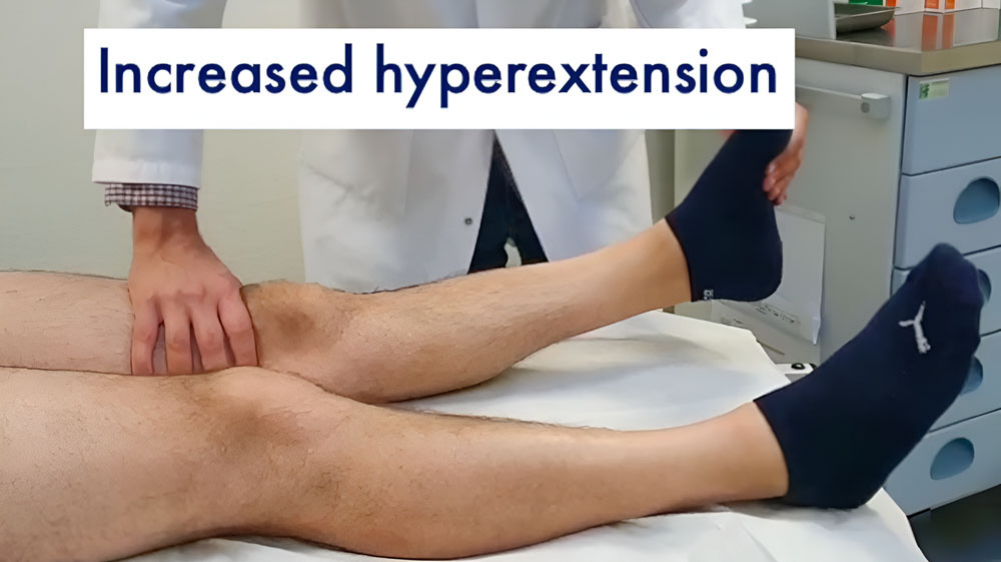
Increased knee hyperextension compared to the contralateral side.

All patients underwent a clinical examination by the senior author (Table 2). Gait appeared to be normal for all patients. A complete assessment of collateral ligaments, posteromedial and posterolateral corner were carried out, including dial test, varus–valgus stress at 0° and 30° and posterior drawer test. All tests were negative, except for the cases identified in Table 2. The injured knees did not display an increased laxity at 90° of flexion. However, an uncommon triad of three signs could be seen with the knee closer to the extension. As shown in the clinical examination video (Online Resource 1), it included a grade 1+ anterior–posterior laxity around 10–20° of knee flexion with a firm endpoint (Figure 1), a grade 1+ tibiofemoral step‐off sign with a posterior drawer at 10–20° of knee flexion (Figure 2) and an increased knee hyperextension compared to the contralateral side (Figure 3).

**Table 2:** Clinical examination and additional investigations and findings.

Patient	1	2	3	4	5
Deformity	None	Varus deformity in both knees	None	None	Varus deformity in both knees
Range of motion	Free	Free	Free	Free	Free
Anterior–posterior laxity (10–20°)	1+	1+	1+	1+	1+
Anterior drawer (90°)	‐	‐	‐	‐	‐
Pivot shift test	‐	‐	‐	‐	‐
Posterior laxity (90°)	‐	‐	‐	‐	‐
Step‐off sign (90°)	‐	‐	‐	‐	‐
Step‐off sign (10–20°)	1+	1+	1+	1+	1+
Hyperextension (compared to contralateral knee)	+5–10°	+3°	>5°	+5°	+3°
Other findings	‐	Lateral collateral ligament no longer palpable and positive dial test at 20° knee flexion	Painful palpation of the posterior horn of the medial meniscus	Dial test positive at 30° of knee flexion	‐
Additional investigation	Bilateral dynamic X‐rays: discrete posterior tibial translation Anteroposterior laxity measurements: SSD 0.2 mm Diagnostic arthroscopy: PCL insufficiency (Grade I/II)	Long leg X‐ray: varus deformity 5° on the right knee and 8.5° on the left knee	Anteroposterior + lateral X‐rays: residual opening of the growth plates	Bilateral dynamic X‐rays: failed to demonstrate a greater laxity in the injured knee	Long leg X‐ray: deformity 3.6° on the right knee and 4.8° on the left knee Anteroposterior laxity measurements: SSD 1.1 mm

Patient	6	7	8	9	10
Deformity	None	None	None	None	None
Range of motion	Free	Free	Free	Free	Free
Anterior–posterior laxity (10–20°)	1+	1+	1+	1+	1+
Anterior drawer (90°)	‐	‐	‐	‐	‐
Pivot shift test	‐	‐	‐	‐	‐
Posterior laxity (90°)	‐	‐	‐	‐	‐
Step‐off sign (90°)	‐	‐	‐	‐	‐
Step‐off sign (10–20°)	1+	1+	1+	1+	1+
Hyperextension (compared to contralateral knee)	+2°	+5°	+2°	+3–5°	+3–5°
Other findings	‐	Positive dial test at 30° of knee flexion	‐	‐	‐
Additional examination	Posterior tibial slope: 13° Anteroposterior laxity measurements: SSD 2.2 mm	‐	Anteroposterior laxity measurements: SSD 0.1 mm	Posterior tibial slope: 8° Closing growth plates Anteroposterior laxity measurements: SSD 0.5 mm	Anteroposterior laxity measurements: SSD 1.2 mm

Patient		11		12	
Deformity		None		None	
Range of motion		Free		Free	
Anterior–posterior laxity (10–20°)		1+		1+	
Anterior drawer (90°)		‐		‐	
Pivot shift test		‐		‐	
Posterior laxity (90°)		‐		‐	
Step‐off sign (90°)		‐		‐	
Step‐off sign (10–20°)		1+		1+	
Hyperextension (compared to contralateral knee)		+0°		Cannot be assessed due to anterior pain	
Other findings		Swollen knee		None	
Additional examination		Antero‐posterior laxity measurements: SSD 0.5 mm		New MRI: consistent with a partial PCL tear	

*Note*: All patients displayed a specific clinical triad (Lachman sign, step‐off sign, hyperextension) close to extension with negative findings at 90° of knee flexion.

Abbreviations: MRI, magnetic resonance imaging; PCL, posterior cruciate ligament; SSD, side‐to‐side difference.

Grade 1+ anterior–posterior laxity around 10–20° of knee flexion with a firm end‐point was the trigger to further evaluate the knee closer to extension as it may be indicative of a pseudo‐Lachman sign rather than a true positive Lachman test. The step‐off sign, usually performed around 90° of knee flexion, was thus reiterated at 10–20° of knee flexion. The grade 1+ magnitude supported the fact that the tibia may be posteriorly displaced between 10° and 20° of knee flexion. In the absence of a positive posterior drawer at 90° and the presence of a higher hyperextension of the knee compared to the contralateral side, the senior author concluded that a partial tear of the PCL and more precisely of the PMB was to be considered. Indeed, the PMB is known to have a primary role in limiting PTT and hyperextension at low knee flexion angles.

### Additional investigations

All MRIs were verified by the senior author and confirmed that there were no findings and no clear signs of damage to the cruciate ligaments. Bilateral dynamic X‐rays were requested for patients 1 and 4. While dynamic X‐ray failed to demonstrate a greater laxity in patient 4, a discrete posterior translation of the tibia could be observed in patient 1. In the latter, a PCL insufficiency grades 1–2 could be confirmed during a diagnostic arthroscopy, which helped to confirm the initial diagnosis made by the senior author during his clinical examination.

Anterior knee laxity measurements using the GNRB® was available for six patients out of 12. The side‐to‐side difference (SSD) at 200 N varied between 0.1 and 2.2 mm, with two patients presenting an SSD above the threshold indicative of an ACL injury of 1.2 mm. However, in the absence of any other clinical sign of an ACL injury, it is plausible that a false positive result might have occurred due to the PMB/PCL tear.

Arthroscopy of eight patients confirmed the pseudo‐Lachman sign with a grade 1+ anterior–posterior laxity at around 10–20° of knee flexion, a normal posterior laxity at 90° of knee flexion and an intact ACL. The following associated injuries were documented: one posterolateral corner injury, three medial meniscus lesions and two lateral meniscus injuries. A non‐anatomic PMB augmentation of the PCL was then performed [11].

## DISCUSSION

The most important finding of this report was the identification of an unknown clinical entity in a series of patients presenting with persistent pain and inability to resume physical activities after knee hyperextension trauma, as well as inconclusive MRIs and clinical examination. In all these patients, an uncommon triad of clinical signs could be observed with the knee close to extension. It consisted in (1) a grade 1+ pseudo*‐*Lachman sign with a firm end‐point, (2) a grade 1+ tibiofemoral step‐off sign with a posterior drawer at 10–20° of knee flexion and (3) a slightly increased knee hyperextension of the injured knee compared to the contralateral side. The combination of patient history, clinical examination and additional investigations led to the assumption that these findings were consistent with a PMB tear of the PCL.

To the authors' knowledge, isolated lesions of the PMB after a knee hyperextension trauma have never been reported. Due to the smaller size and inferior strength of the PMB compared to the ALB [2], the role of the former has for long been neglected and only recently regained popularity thanks to Paschos who reminded that ‘PMB is small but vital to the PCL biomechanics’ [19]. The PMB is known to be tight close to extension. Its position and orientation places it in a good position to be a secondary restraint of PTT at low degrees of knee flexion and of knee hyperextension [8]. As for the ALB, it is slack in full extension which prevents the structure from being injured during hyperextension traumas, at least to some degree. It is however tight above 40° of knee flexion where it is the primary restraint of PTT [6]. Considering these biomechanical properties, a tear to the PMB may thus have the following impact: no increased PTT above 40° of knee flexion, slight increase of PTT below 40° of knee flexion and small increase of hyperextension. This corresponds to the uncommon clinical triad observed in the 12 patients of this original case series.

In the present case report, the standard clinical examination of the PCL at 90° of knee flexion did not show any abnormalities, suggesting that the ALB of the PCL remained uninjured. The 1+ pseudo‐Lachman and the 1+ tibiofemoral step‐off signs observed at 20° of knee flexion highlight a small but significant posterior translation at a low flexion angle which would be consistent with an isolated lesion of the PMB of the PCL. These findings are also in agreement with previous cadaver studies investigating isolated sectioning of the PMB [10, 14]. Markolf et al. [14] reported a small but significant increase in PTT after isolated PMB sectioning of 1.06 mm at 0° of knee flexion. The increase only remained significant at 10° of knee flexion and could not be observed from 30° to 90° of knee flexion. Kennedy et al. [11] later confirmed that the codominant relationship between ALB and PMB suggests that only complete PCL tears can lead to an excessive PTT and that the increase of PTT in partial tears is probably minimal. It thus appears that partial PCL injuries may be much more difficult to identify during clinical examination than complete PCL ruptures.

The authors believe that this new clinical entity has frequently been unrecognised given the lack of diagnostic resources to formally identify it. While the diagnostic strategy highlighted in this study remains clinical, further investigations are needed to determine whether MRI may help orientating the diagnosis of PMB tears of the PCL. While MRI is known to be limited to directly identify an isolated or partial PCL injury [18, 20], some indirect signs such as bone marrow oedema may provide some information to look for these injuries. For example, Ali et al. [1] reported that the presence of anterolateral tibial plateau oedema following knee hyperextension injuries was strongly associated with the presence of a PCL injury (odds ratio = 26.0, *p* = 0.003). The authors speculated that this observation could be caused by a reverse pivot shift mechanism (i.e., internal rotation and varus hyperextension), leading to an impact on the anterolateral tibial plateau. Unfortunately, they did not further investigate which bundles were involved in the reported injuries. A strong effort is thus still needed to be able to properly recognise partial tears of the PCL. This is all the most important as both the literature [2, 3, 6, 8, 11, 14] and the present findings confirm that patients with partial PCL tears present with unspecific, subacute or chronic symptoms of pain and swelling and, most of the time, inconclusive MRIs.

It is important to highlight that the present authors performed additional investigations to rule out other possible soft tissue damages within the knee. The arthroscopy of eight patients helped to confirm that the ACL was intact in these knees. While some associated lesions were documented (one posterolateral corner injury, three medial meniscus lesions and two lateral meniscus injuries), none of them was systematically observed nor could it explain, according to our current knowledge on knee biomechanics [8], the presence of the systematic clinical triad as described in the present paper in every patient. It may be argued that the POL, which has been reported to have a greater role than the PMB to restrain PTT and hyperextension [8, 16], may be injured here. In a recent study published by Noyes et al. [17], the authors found that the PCL represents 12.9% of the resisting force to knee hyperextension, while the POL accounts for only 7.7%. In this study, the posteromedial capsule and the posterior oblique ligament were the main structures resisting hyperextension (21.7%). This study shows that the main restrictors of hyperextension are multiple soft tissue, with no single dominant. But if we look only at the POL, and compare it with the PCL, the PCL provides an increased resisting moment to hyperextension. Therefore, it is reasonable to believe that an injury to this structure may not be capable of causing the triad of clinical symptoms related in the present case series. Moreover, in the present study, neither in the MRI nor in the clinical or arthroscopic examination, any lesions to those ‘posterior capsular structures’ were found

The main limitation of the present study is the limited number of patients. To the best of the authors' knowledge, this case series is the first to describe this clinical entity. In a recent editorial, Paschos et al. [19] highlighted again the role of the PMB, particularly in double‐bundle PCL reconstructions. The authors suggested that a PMB reconstruction may provide additional resistance to PTT at lower degrees of knee flexion [10]. This provided the rationale to the recently described isolated augmentation [9] of the PMB which may be a therapeutic strategy in partial PCL injuries which are limited to the PMB. This has, however, still to be demonstrated in further studies.

## CONCLUSION

Clinical and arthroscopic findings revealed an increased posterior laxity close to knee extension which normalised at 90° of knee flexion, as well as an increased hyperextension of the injured knee compared to the contralateral side. These findings are consistent with a PMB tear of the PCL, the PMB being tight in extension in opposition to the ALB. In patients with a history of hyperextension knee trauma, unresolved persistent pain and inability to resume physical activities, clinicians should consider extending their investigations with the knee close to extension to identify this clinical entity.

## AUTHOR CONTRIBUTIONS

Caroline Mouton, Maximiliano Ibañez and Felix Hoffmann have made substantial contributions to conception, study design and acquisition/interpretation of data and drafting the manuscript. Caroline Mouton, Romain Seil and Joan Carles Monllau have been involved in drafting or revising the manuscript critically. Each author has given final approval of the version to be published and agrees to be accountable for all aspects of the work in ensuring that questions related to the accuracy or integrity of any part of the work are appropriately investigated and resolved.

## CONFLICTS OF INTEREST STATEMENT

Caroline Mouton is chairwoman of the European Society for Sports Traumatology, Knee Surgery and Arthroscopy (ESSKA) Basic Science Committee and editorial board for *Knee Surgery, Sports Traumatology, Arthroscopy* (*KSSTA*) and *Journal of Experimental Orthopaedics*. Prof. Joan Carles Monllau reports a relationship with Smith and Nephew Inc. and Conmed that includes consulting or advisory. He is vice president of ESSKA; past president of Spanish Arthroscopy Association; and editorial board member of *Arthroscopy*. Prof. Romain Seil reports a relationship with Smith and Nephew Inc., Olympus Corporation and Amplitude Ventures that includes consulting or advisory. Reports a relationship with Virtamed that includes funding grants. He is president of Luxembourg Institute of Research in Orthopedics, Sports Medicine and Science; past president of Society for Orthopaedic Traumatologic Sports Medicine (GOTS; Gesellschaft für Orthopädisch‐Traumatologische Sportmedizin)/ESSKA, Chairman of THE MENISCUS 2022; Vice Chairman of ESSKA meniscus certification module and editorial board member of *KSSTA*, *Arthroscopy* and *Sports Orthopaedics and Traumatology* (*SOT*). The remaining authors declare no conflict of interest.

## ETHICS STATEMENT

As the study was performed in accordance with ethical standards of the institutional and national research committees, it did not require prior approval. The data for this case series study were collected from the medical records of patients which were obtained for routine clinical purposes. All data were exported anonymously by the team of clinicians taking care of the included patients who therefore were granted access to their medical records.

## Supporting information

Supporting information.

## Data Availability

The data sets generated and analysed during the current study are available at the Centre Hospitalier Luxembourg, CHL Eich repository. Data is accessible upon request. The data includes Excel files containing the measurements of knee injuries, the MRI images in DICOM format and statistical analysis scripts in R. For additional information or data requests, please contact Prof. Romain Seil at rseil@yahoo.com.
